# Effectiveness of single-volume continuous jogging and intermittent running on short-term blood glucose in elderly patients with type 2 diabetes mellitus

**DOI:** 10.3389/fmed.2025.1587597

**Published:** 2026-01-12

**Authors:** Yan Lv, Yue-Mei Wu, Li-Li Wang, Li-Li Yan, Lin Ge, Xun Du, Jing-Juan Liu

**Affiliations:** 1 Medical Examination Center, Taihu Sanatorium of Jiangsu Province, Wuxi, Jiangsu Province, China; 2 Nursing Department, Taihu Sanatorium of Jiangsu Province, Wuxi, Jiangsu Province, China; 3 Health Promotion Center, Taihu Sanatorium of Jiangsu Province, Wuxi, Jiangsu Province, China; 4 Health Resort, Taihu Sanatorium of Jiangsu Province, Wuxi, Jiangsu Province, China

**Keywords:** blood glucose, continuous jogging, elderly, intermittent running, type 2 diabetes mellitus

## Abstract

**Objective:**

To investigate the effects of different single-volume exercise modalities on physiological indicators, including blood glucose, heart rate (HR), and fatigue, in elderly patients with type 2 diabetes mellitus (T2DM). Additionally, this study assessed participants’ subjective perceptions and acceptance of these exercises to inform the development of reasonable and effective exercise prescriptions.

**Methods:**

Thirty elderly patients with T2DM participated in this self-controlled, pre-post intervention study. Experiments were conducted over three separate days: two exercise days involving continuous jogging (CJ) and intermittent running (IR), with the sequence randomized, and one control day without exercise. Participants in the CJ group were instructed to run at a self-perceived moderate intensity. Based on pre-test results, running speed was set between 4–6 km/h for approximately 20–30 min. In the IR group, participants ran at a preset speed of 6–8 km/h for 4 min, followed by 3 min of stationary rest, repeating this cycle throughout the session. Blood glucose was measured at fasting, pre-exercise, immediately post-exercise, at 11:00 a.m. (before lunch), and at 4:00 p.m. Blood glucose data were analyzed using two-way repeated measures ANOVA, followed by one-way ANOVA with *post hoc* tests to identify specific differences. HR and rating of perceived exertion (RPE) were analyzed using one-way ANOVA, while blood pressure and PACES scores were compared using paired *t*-tests.

**Results:**

Participants had a mean age of 68.20 ± 8.13 years, diabetes duration of 8.42 ± 5.07 years, and body mass index (BMI) of 24.46 ± 3.54 kg/m^2^. Blood glucose levels decreased immediately after exercise in both modalities and were significantly lower than pre-exercise values. At 11:00 a.m., blood glucose was significantly lower in the exercise groups compared to the control group, whereas at 4:00 p.m., no significant differences were observed among the groups. No differences in blood glucose levels between CJ and IR were identified at any time point. Immediately post-exercise, HR reached 73% of the maximum in the CJ group and 84% in the IR group. There were no significant differences in RPE scores between the exercise modalities.

**Conclusion:**

CJ and IR had similar effects on short-term blood glucose regulation. However, participants demonstrated greater acceptance of IR, suggesting it may be more suitable for elderly patients with T2DM.

## 1 Introduction

Numerous studies have demonstrated that exercise is an effective intervention for individuals with type 2 diabetes mellitus (T2DM) (1). Exercise reduces diabetes incidence, lowers blood glucose and glycosylated hemoglobin levels, improves pancreatic *β*-cell function, regulates blood lipid levels, and alleviates diabetes-related complications (2).

China currently has the highest number of diabetes cases worldwide, with approximately 35.5 million elderly individuals affected by T2DM, representing nearly one-quarter of the global elderly diabetic population (3). This number continues to increase (4). Elderly T2DM patients frequently present with more comorbidities, atypical symptoms, and a higher risk of hypoglycemia (5). Consequently, many patients hesitate to engage in moderate- or high-intensity exercise due to safety concerns, despite having adequate physical function (6, 7). Additionally, elderly patients encounter practical limitations arising from age-related declines in physical function and difficulties in maintaining long-term adherence to prescribed exercise regimens (8).

Recent studies have confirmed the safety and effectiveness of high-intensity exercise in patients with T2DM (9). High-intensity interval training effectively reduces fasting and postprandial blood glucose and enhances skeletal muscle metabolism (10). Specifically, high-intensity interval training increases GLUT-4 expression, promotes glucose transport, and elevates blood lipocalin levels in elderly T2DM patients (11). Additionally, it lowers cholesterol and fatty acid synthesis, reduces lipid deposition, minimizes lipid toxicity to insulin-producing cells, and improves insulin sensitivity (12, 13). Compared to traditional exercise interventions, high-intensity interval training achieves similar or greater improvements in blood glucose control while significantly reducing total exercise duration (14, 15).

High-intensity interval exercise modalities are increasingly promoted; however, their acceptance among elderly T2DM patients remains uncertain. Managing elderly patients with T2DM involves unique challenges, such as age-related declines in exercise tolerance, high prevalence of comorbidities, and elevated concerns regarding exercise-associated risks, including hypoglycemia (14). These factors significantly compromise the feasibility and long-term adherence to traditional exercise regimens (13). Thus, investigating the acute physiological effects of single-bout exercise is critical. A time-efficient exercise modality may be more practical and sustainable for this population compared to prolonged or complex training programs. Therefore, this study aimed to examine the acute physiological effects of different single-bout exercise modalities in elderly patients with T2DM to provide empirical evidence for developing precise, feasible, and safe exercise prescriptions. Additionally, the study assessed participants’ subjective experiences and acceptance of these exercises to inform practical and effective exercise prescriptions for elderly T2DM patients.

## 2 Objectives and methods

### 2.1 Objectives

A convenience sampling approach was employed to select 30 elderly patients with T2DM who attended the Department of Endocrinology at Taihu Sanatorium, Jiangsu Province, between December 2024 and February 2025.

#### 2.1.1 Inclusion criteria

① Diagnosed according to the *Chinese Guidelines for the Prevention and Control of Type 2 Diabetes Mellitus* (2022 edition) (7): diabetic symptoms combined with at least one of the following criteria: random plasma glucose ≥11.1 mmol/L, fasting plasma glucose ≥7.0 mmol/L, or 2-h postprandial plasma glucose ≥11.1 mmol/L in the Oral Glucose Tolerance Test (OGTT). ② Age ≥60 years.

#### 2.1.2 Exclusion criteria

Patients with contraindications to exercise were excluded, including but not limited to: ① Severe complications involving the heart, brain, kidneys, eyes, feet, or nervous system (e.g., proliferative retinopathy, kidney disease stage IV or higher, severe cardiovascular and cerebrovascular diseases, autonomic neuropathy). ② Malignant tumors. ③ Inability to tolerate moderate-intensity exercise. ④ Resting heart rate (HR) exceeding 120 beats/min or blood pressure above 160/100 mmHg. ⑤ Fasting blood glucose >16.7 mmol/L. ⑥ Diabetic foot or severe joint disorders. ⑦ Acute diabetic complications (e.g., diabetic ketoacidosis, lactic acidosis). After initial screening, 30 elderly patients with T2DM who met the inclusion criteria underwent medical examinations to exclude exercise contraindications. All participants were fully informed of the study objectives, procedures, and potential risks. They voluntarily agreed to participate and provided written informed consent before enrollment.

### 2.2 Experimental procedures and test indicators

#### 2.2.1 Sociodemographic characteristics and biomarker records

Height, weight, body mass index (BMI), and HR were recorded for all enrolled patients. Additionally, glycosylated hemoglobin, C-peptide, insulin, and triglycerides were assessed using venous blood samples.

#### 2.2.2 Dietary arrangements and daily activities

Patients whose medication regimens and clinical conditions remained stable during the 72 h following admission and who met inclusion criteria were provided diets designed by endocrinologists according to BMI and physical activity. Participants were required to follow their prescribed dietary plans. During the 3 days of exercise intervention, breakfast and lunch were standardized, prepared, and provided by the Nutrition Department. All participants received a standardized diet supplying 25–30 kcal/kg ideal body weight per day, with carbohydrates comprising 50% of total energy. To prevent interference with study results, strong tea, coffee, and sugary beverages were prohibited. Throughout the experimental period, patients maintained a regular daily schedule and refrained from activities other than those specified. Efforts were made to minimize exposure to mental stressors, insomnia, and other factors that could influence physiological responses. Unless medically necessary, patients’ glucose-lowering medication regimens remained unchanged to minimize their potential impact on study outcomes.

### 2.3 Exercise implementation program

#### 2.3.1 Overall design of exercise experiments

This study employed a cross-over design, where each participant completed both CJ and IR exercises. Experiments were conducted over three separate days, including two exercise days (CJ day and IR day) and one control day without exercise. The sequence of exercise modalities was randomized using the sealed envelope method. Specifically, a research assistant uninvolved in data collection placed each exercise program into sequentially numbered, opaque sealed envelopes based on a computer-generated random sequence. Participants drew envelopes, and the principal investigator opened them sequentially to assign interventions. The control day served as a baseline, with all other conditions (diet, treatment, and daily routine) identical to exercise days. To reduce the influence of prior exercise, a minimum interval of 3 days separated experimental sessions. Additionally, the order of the three experimental days was randomized among participants to prevent order-related biases.

Before the formal experiments, participants underwent an acclimatization training session, which involved running 500 meters daily for three consecutive days. No specific requirements regarding exercise duration or speed were imposed; participants were encouraged to run at a comfortable pace. This training ended 3 days before the main experiment began.

**Figure 1:**
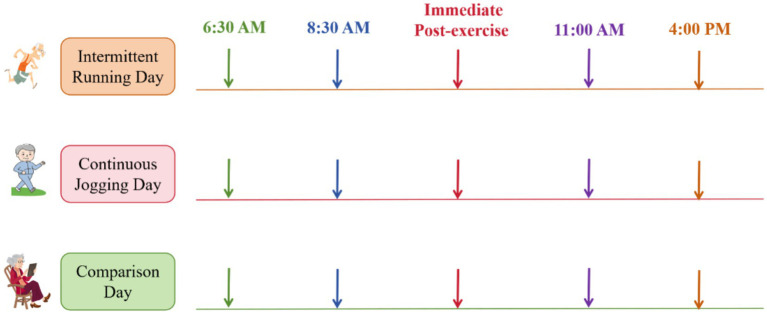
Exercise grouping and testing procedures (arrows indicate blood glucose measurement times).

On both exercise and control days, fasting blood glucose was measured at 6:30 a.m., immediately followed by a standardized meal for all participants. On exercise days, participants began exercising 2 hours after breakfast. Blood glucose was measured before exercise (Pre, approximately 2 hours after breakfast), immediately after exercise (Post), at 11:00 a.m., and at 4:00 p.m. On the control day, measurements occurred at identical times, except for the immediate post-exercise reading (Figure 1). Investigators performed all blood glucose measurements using capillary blood samples. Measurements were obtained using a Roche ACCU-CHEK Performa glucose meter and Accu-Chek Softclix test strips (Batch No. 471826). Calibration of the glucose monitoring system involved two steps: (1) automatic electronic calibration using a coded chip specific to each test strip lot, and (2) daily biochemical verification with manufacturer-supplied control solutions (50 mg/dL, 120 mg/dL, and 300 mg/dL). Calibration was considered acceptable only if all control measurements fell within the manufacturer’s specified range.

#### 2.3.2 Specific requirements for exercise programs

In the CJ group, participants maintained a running pace at a perceived moderate intensity. Based on pre-test results, the target speed was set at 4–6 km/h, lasting approximately 20–30 min. Participants jogged continuously throughout the session and were instructed to avoid fast walking.

In the IR group, participants ran at a speed of 6–8 km/h. Each interval included 4 min of running followed by 3 min of stationary rest. This cycle was repeated four times. High-intensity exercise was defined as intermittent aerobic activity performed at 80–89% of HR reserve (12). This protocol consisted of a total running duration of 16 min and rest duration of 9 min, resulting in an overall session length of 25 min. This duration aligned with the intended session time range of 24–32 min.

Throughout the exercises, researchers closely monitored and recorded each participant’s distance and duration.

### 2.4 Monitoring of exercise intensity

#### 2.4.1 HR

Participants wore HR monitors during exercise, with measurements recorded every 500 meters. HRs were specifically measured at pre-exercise (HR-0), 0.5 km (HR-1), 1 km (HR-2), 1.5 km (HR-3), and 2 km (HR-4).

#### 2.4.2 Rating of perceived exertion (RPE)

The RPE scale ranges from 6 to 20, with higher scores indicating greater subjective fatigue. During the exercise sessions, participants reported their exertion levels at 500-meter intervals. RPE values were recorded at 0.5 km (RPE-1), 1 km (RPE-2), 1.5 km (RPE-3), and 2 km (RPE-4).

### 2.5 Activity enjoyment

The Physical Activity Enjoyment Scale (PACES) was used for scoring enjoyment (16). PACES comprises 18 items, each scored from 1 to 7, with a maximum total of 126 points. The scale includes items requiring reverse scoring, which were converted positively when calculating total scores. Higher scores indicate greater enjoyment of the activity. Participants completed the PACES questionnaire immediately after exercise, based on their own experiences.

### 2.6 Exercise safety monitoring

Before exercise, participants were educated about the benefits of physical activity and informed of potential adverse reactions or injuries during exercise. Researchers assisted in checking participants’ feet, shoes, and socks to ensure proper fit and safety prior to each session. Throughout the exercise, both participants and researchers carried candy to manage potential hypoglycemic episodes. Researchers closely monitored participants during exercise to promptly identify adverse reactions and provided immediate medical assistance when necessary.

#### 2.6.1 Exercise cessation criteria

Exercise was immediately stopped if participants experienced falls, injuries, hypoglycemia, dizziness, nausea, palpitations, chest pain, or other discomfort. Additionally, exercise was terminated if participants reported that the intensity exceeded their tolerance, felt extremely fatigued, or were unable or unwilling to continue.

## 3 Statistical analysis

Statistical analyses were performed using SPSS 21.0. Results are expressed as Mean ± SD (X̄ ± S). Two-way repeated measures ANOVA was used to analyze blood glucose differences between and within groups. When significant differences were identified, one-way ANOVA with *post hoc* tests was conducted to determine specific differences. HR and RPE values were analyzed using one-way ANOVA, while blood pressure and PACES scores were compared using paired t-tests. A *p*-value <0.05 was considered statistically significant.

## 4 Results

### 4.1 General information

A total of 30 elderly T2DM patients participated in the study. All patients received insulin therapy, with 18 patients also taking oral medication. The general characteristics and basic biomarkers of patients are presented in Table 1.

**Table 1:** General information and biomarker.

Items	(X_ ± S)
Age (years)	68. 20 ± 8.13
Disease duration (years)	8.42 ± 5.07
BMI (kg/m^2^)	24.46 ± 3.54
Glycosylated hemoglobin (%)	9.27 ± 3.05
C-peptide (ng/mL)	2.38 ± 1.08
Insulin (mIU/mL)	6.59 ± 4.83
Triglyceride (mmol/L)	2.32 ± 2.24
Cholesterol (mmol/L)	4.56 ± 1.41

### 4.2 Exercise duration and speed

Exercise duration and speed for the CJ and IR groups are detailed in Table 2. Results showed that actual running speed during active intervals was higher in the IR group than in the CJ group. Considering prescribed rest intervals, total session duration was also longer in the IR group. However, because rest periods were included, overall average speed (total distance divided by total session duration) was consequently lower in the IR group compared to the CJ group.

**Table 2:** Exercise time and speed of the continuous jogging and intermittent running groups.

Items	Continuous jogging group (X_ ± S)	Interval running group (X_ ± S)
Exercise duration (min)	20.13 ± 1.81	17.53 ± 1.64
Break (min)	0	11.52 ± 3.04
Total exercise time (min)	20.13 ± 1.81	27.82 ± 3.02
Actual speed (km/h)	5.86 ± 0.56	6.71 ± 0.66
Average speed (km/h)	5.86 ± 0.56	4.08 ± 0.54

### 4.3 HR

HR measurements at different time points for the CJ and IR groups are shown in Table 3. HR values (HR-0 to HR-4) represent resting HR (HR-0) and measurements at 0.5 km (HR-1), 1 km (HR-2), 1.5 km (HR-3), and 2 km (HR-4). Results indicated no significant difference between groups in pre-exercise HR (HR-0) (*p* > 0.05). However, during exercise (HR-1 to HR-4), HRs were significantly higher in the IR group than in the CJ group (*p* < 0.05). Immediately after exercise, HRs reached 73.4 ± 4.6% and 84.0 ± 4.2% of maximum HR in the CJ and IR groups, respectively. Within-group comparisons showed HR at HR-1 was significantly higher than at HR-0 in both groups (*p* < 0.05). No significant difference occurred between HR-1 and HR-4 within either group (*p* > 0.05).

**Table 3:** Heart rate (beats/min) at different stages in the continuous jogging and intermittent running groups.

HR	Continuous jogging group (X_ ± S)	Interval running group (X_ ± S)	*t*	*p*
HR-0	80.33 ± 8.34	80.93 ± 8.53	−0.324	0.767
HR-1	125.47 ± 8.69^*^	141.24 ± 8.43^*,#^	−7.647	<0.001
HR-2	127.67 ± 8.56^*^	142.32 ± 8.38^*,#^	−6.675	<0.001
HR-3	128.53 ± 9.68^*^	143.23 ± 8.89^*,#^	−6.878	<0.001
HR-4	129.07 ± 8.76^*^	144.12 ± 8.79^*,#^	−7.435	<0.001
*F*	147.543	265.634		
*p*	<0.001	<0.001		

Comparison within groups: ^*^p < 0.05 compared with HR-0.

Comparison between groups: ^#^*p* < 0.05 compared with the continuous jogging group.

### 4.4 Blood glucose

As indicated in Tables 4–6, comparisons among groups showed no significant differences in fasting and pre-exercise blood glucose (*p* > 0.05). Immediate post-exercise blood glucose levels were also not significantly different between CJ and IR groups (*p* > 0.05). At 11 a.m., blood glucose was significantly lower in the exercise groups compared to the control group (*p* < 0.05), but no significant difference was found between CJ and IR groups (*p* > 0.05). No significant differences were found among groups at 4 p.m. (*p* > 0.05). Within-group comparisons showed pre-exercise blood glucose was significantly higher than fasting levels in all three groups (*p* < 0.05). Immediate post-exercise blood glucose was significantly lower than pre-exercise levels in both exercise groups (*p* < 0.05) but remained higher than fasting levels (*p* < 0.05). Blood glucose at 11 a.m. was significantly lower than pre-exercise levels in all three groups (*p* < 0.05), but not significantly different from immediate post-exercise levels (*p* > 0.05). Blood glucose at 4 p.m. showed no significant difference compared to 11 a.m. in any group (*p* > 0.05).

**Table 4:** Blood glucose (mmol/L) at different time points in different experimental groups.

Groups	Fasting (X_ ± S)	Pre (X_ ± S)	Post (X_ ± S)	11 a.m. (X_ ± S)	4 p.m. (X_ ± S)	*F*	*p*
Comparison group	6.67 ± 1.42	12.75 ± 3.43^a^	NA	8.79 ± 2.02^bd^	9.13 ± 2.43^ad^	27.582	<0.001
Continuous jogging group	6.53 ± 1.56	12.73 ± 3.14^a^	9.83 ± 3.23^bc^	7.56 ± 2.14^1d^	8.78 ± 2.67^d^	18.807	<0.001
Interval running group	6.83 ± 1.72	12.83 ± 3.24^a^	9.79 ± 3.01^ac^	7.41 ± 1.78^1d^	8.98 ± 2.42^d^	20.435	<0.001
*F*	0.046	0.072	1.103	3.872	0.593		
*p*	0.955	0.93	0.314	0.047	0.612		

Comparison between different groups: ^1^*p* < 0.05 compared with control group.

Comparison between different time points: ^a^*p* < 0.01 compared with fasting; ^b^*p* < 0.05 compared with fasting; ^c^*p* < 0.05 compared with Pre; ^d^*p* < 0.01 compared with Pre.

NA, Not measured.

**Table 5:** Comparison of blood glucose at the same time point among different groups (*p*-value).

Items	Fasting	Pre	Post	11 a.m.	4 p.m.
Comparison vs. continuous jogging	0.895	0.882	NA	0.041^*^	0.412
Comparison vs. interval running	0.884	0.874	NA	0.034^*^	0.443
Continuous jogging vs. interval running	0.882	0.802	0.312	0.895	0.813

^*^p < 0.05; NA, Not measured.

**Table 6:** Comparison of blood glucose at different time points within the same group (*p*-value).

Time point	Items	Comparison group	Continuous jogging group	Interval running group
Fasting vs.	Pre	<0.001^*^	<0.001^*^	<0.001^*^
	Post	NA	0.023^*^	0.013^*^
	11 a.m.	0.021^*^	0.864	0.892
	4 p.m.	0.032^*^	0.712	0.293
Pre vs.	Post	NA	0.034^*^	0.023^*^
	11 a.m.	<0.001^*^	<0.001^*^	<0.001^*^
	4 p.m.	<0.001^*^	<0.001^*^	<0.001^*^
Post vs.	11 a.m.	NA	0.223	0.213
	4 p.m.	NA	0.813	0.892
11 a.m. vs.	4 p.m.	1.082	0.897	0.983

^*^p < 0.05; NA, Not measured.

### 4.5 RPE scores

RPE scores at different time points for the CJ and IR groups are presented in Table 7. The values (RPE-1 to RPE-4) correspond to measurements taken at 0.5 km, 1 km, 1.5 km, and 2 km, respectively. Between-group comparisons showed that RPE-1 scores were significantly lower in the CJ group than in the IR group (*p* < 0.05). No significant differences were found between the groups from RPE-2 to RPE-4 (*p* > 0.05). Within-group comparisons revealed that RPE scores increased progressively in both groups, with each successive time point significantly higher than the previous one (*p* < 0.05).

**Table 7:** RPE scores of the continuous jogging and intermittent running groups.

RPE	Continuous jogging group (X_ ± S)	Interval running group (X_ ± S)	*t*	*p*
RPE-1	12.03 ± 1.09	13.53 ± 1.89	−5.201	<0.001
RPE-2	14.88 ± 2.04^*^	14.43 ± 2.01^*^	−0.402	0.889
RPE-3	15.23 ± 2.73^*,#^	16.02 ± 2.21^*,#^	−0.103	0.887
RPE-4	17.03 ± 1.85^*,#,†^	18.43 ± 1.98^*,#,†^	−0.612	0.727
*F*	45.223	26.768		
*p*	<0.001	<0.001		

*p < 0.05 compared with RPE-1; ^#^*p* < 0.05 compared with RPE-2; ^†^*p* < 0.05 compared with RPE-3.

### 4.6 Paces

As shown in Table 8, PACES scores were significantly higher in the IR group compared to the CJ group (*p* < 0.05).

**Table 8:** Post-exercise PACES of the continuous jogging and intermittent fast running groups.

Item	Continuous jogging group	Interval running group	*t*	*p*
PACES	78.6 ± 2.03	91.3 ± 1.27	−21.453	<0.001*

^*^p < 0.05.

## 5 Discussion

In this study, 30 elderly T2DM patients participated in two exercise modalities (2 km of CJ and IR) to assess their effects on blood glucose, HR, and subjective perceptions. Time points (11:00 a.m. and 4:00 p.m.) were selected to represent key phases of daytime glucose metabolism. Specifically, 11:00 a.m. reflects the early post-lunch prandial state, while 4:00 p.m. corresponds to the late postprandial phase, a period characterized by potential declines in insulin sensitivity. This approach allowed evaluation of glucose homeostasis during a clinically relevant period of afternoon glucose instability. The results showed that both exercise modalities, performed 2 hours after breakfast, significantly reduced blood glucose immediately post-exercise compared to pre-exercise levels. At 11:00 a.m., blood glucose was significantly lower in both exercise groups than in the control group. However, at 4:00 p.m., no significant differences in blood glucose were observed among the three groups. Additionally, blood glucose levels did not differ significantly between the two exercise modalities at any measured time point.

### 5.1 Acute glycemic response and exercise energy metabolism

This study found that blood glucose decreased significantly immediately after both single-bout CJ and IR exercises, with no statistical difference between the two groups. This result may stem from the similar total energy expenditure under the same exercise distance. Research indicates that the energy cost of running per unit distance remains relatively constant and is not significantly influenced by speed (17, 18). In this study, although the IR group achieved a higher heart rate during exercise intervals (84% of maximum heart rate), falling into the high-intensity category, while the CJ group was at moderate-to-high intensity (73%), when exercise intensity exceeds 60% of maximal oxygen uptake, carbohydrates become the primary energy source for muscle metabolism (19). According to ACSM guidelines, both exercise modalities in this study, estimated based on heart rate, exceeded this threshold (20). Therefore, under equal exercise volume, both modalities likely utilized similar proportions of glucose and lipids, leading to comparable reductions in blood glucose (2). A recent 12-week aerobic exercise intervention study controlling for total energy expenditure also reported no significant differences in fasting blood glucose and HbA1c between continuous and intermittent exercise groups in T2DM patients (21), supporting the findings of the present study regarding the acute effects of a single exercise session.

### 5.2 Duration of post-exercise glycemic effects and physiological rhythms

At 2 hours post-exercise (11:00 a.m.), blood glucose in the exercise groups remained significantly lower than in the control group, suggesting that the glycemic benefits of a single exercise session can persist for a period. This may be related to excess post-exercise oxygen consumption and sustained improvements in insulin sensitivity (22). However, this effect did not extend to 4:00 p.m., by which time no differences in blood glucose were observed among the three groups. This may result from the dynamic interaction of several physiological factors: the new glycemic load introduced by lunch, the natural decay over time of the insulin-sensitizing effects from the morning exercise, and the inherent circadian rhythm of glucose metabolism—often characterized by decreased insulin sensitivity and increased hepatic glucose output in the afternoon. Thus, the absence of sustained improvement at 4:00 p.m. does not indicate ineffectiveness of the exercise modalities. Instead, it reflects the interaction between the acute exercise stimulus and ongoing metabolic rhythms, highlighting the temporal limits of exercise-induced glycemic control.

### 5.3 Perceived exertion, exercise enjoyment, and clinical feasibility

Although the IR group achieved higher exercise intensity, there was no significant difference in post-exercise ratings of perceived exertion (RPE) between the two groups. Discussions revealed that participants generally perceived the interspersed rest periods in the IR protocol as providing psychological and physiological relief, making subsequent running segments feel easier to complete, despite accumulated fatigue towards the end. This structure may reduce the psychological burden associated with continuous exercise. More importantly, the IR group scored significantly higher than the CJ group on the Physical Activity Enjoyment Scale (PACES). The characteristic of intermittent exercise—combining short bursts of effort with rest—may offer greater psychological relaxation, thereby enhancing the exercise experience and enjoyment (23). For elderly T2DM patients with declining exercise tolerance and multiple comorbidities, exercise enjoyment is a critical factor influencing long-term adherence (24, 25). Therefore, although the two modalities were equivalent in terms of acute glycemic regulation, IR may offer greater feasibility and sustainability in clinical practice due to its better subjective acceptability and experience.

## 6 Conclusion

Single-volume exercise offers patients greater flexibility in time management, making it highly valuable for daily life. Both CJ and IR showed similar effects on blood glucose regulation. However, elderly T2DM patients expressed greater enjoyment with IR, suggesting it as a more practical and preferable choice. This preference, combined with the inherent flexibility of IR, provides significant practical value for exercise recommendations. Therefore, we propose IR as a viable and potentially more sustainable alternative to traditional continuous exercise for personalized exercise prescriptions in elderly T2DM patients. From a public health perspective, promoting IR could effectively enhance long-term exercise adherence and improve glycemic control in this population.

### 6.1 Limitations

This study serves as a preliminary investigation laying the groundwork for future large-sample research. Given that elderly patients with diabetes are often characterized by multiple comorbidities and clinical complexity, the current study is limited by its relatively small sample size, which precluded in-depth subgroup analyses based on different clinical characteristics (e.g., varying limb function, types of comorbidities). Future studies plan to expand the sample size to systematically examine the specific differences in glycemic responses to different single-session exercise modalities among elderly T2DM patients with diverse clinical profiles.

## Data Availability

The raw data supporting the conclusions of this article will be made available by the authors, without undue reservation.
